# Efficacy and Safety of Dupilumab in the Treatment of Hand Eczema: A Retrospective Study

**DOI:** 10.3390/jcm13071876

**Published:** 2024-03-24

**Authors:** Claudia Paganini, Virginia Maffei, Laura Vellucci, Marina Talamonti, Alessandra Petruzzellis, Lorenzo Le Pera, Cosimo Di Raimondo, Luca Bianchi, Marco Galluzzo

**Affiliations:** 1 Department of Systems Medicine, University of Rome “Tor Vergata”, 00133 Rome, Italy; cld.paganini@gmail.com (C.P.); virginiamaffei1@gmail.com (V.M.); vellucci.laura@gmail.com (L.V.); alessandra.petruzzellis@gmail.com (A.P.); dott.lorenzo.lepera@gmail.com (L.L.P.); luca.bianchi@uniroma2.it (L.B.); 2 Dermatology Unit, Fondazione Policlinico Tor Vergata, 00133 Rome, Italy; talamonti.marina@gmail.com (M.T.); cosimodiraimondo@gmail.com (C.D.R.)

**Keywords:** hand eczema, dupilumab, chronic hand eczema, atopic dermatitis

## Abstract

**Background:** Hand eczema (HE) is a prevalent chronic condition that exerts a substantial and enduring adverse effect on quality of life (QoL) and imposes an economic burden on society. Managing HE poses challenges due to the limited effectiveness and potential adverse effects associated with many currently available topical and systemic treatments. **Methods:** This article examines twenty-one patients affected by HE treated with dupilumab, a fully human monoclonal antibody targeting interleukin IL-4 and IL-13 signaling. This involves a retrospective descriptive statistical analysis. **Results:** At week 6, HECSI-75 was achieved by 12 patients (57.9%). The proportion of patients meeting the HECSI-75 criteria steadily increased over the observation weeks, reaching 90% at week 16 and 100% at week 104. Furthermore, HECSI-90 and HECSI-100 were achieved by 75% and 60% of patients at week 16 and by 100% and 85% of patients at week 68, respectively. All patients who reached week 104 maintained complete disease remission according to HECSI 100. **Conclusions:** In all patients, dupilumab was shown to be an effective drug in achieving disease clearance, as indicated by all the parameters considered at each evaluation point (Week 6, Week 16, Week 32, Week 52, Week 68, Week 84, and Week 104), in comparison to the initial baseline.

## 1. Introduction

Hand eczema (HE) is a chronic inflammatory condition that affects approximately 10% of the general population, with a higher prevalence among individuals in high-risk occupations such as healthcare workers, hairdressers, cooks, cleaners, and others who are consistently exposed to water and chemicals [1]. It is characterized by various clinical manifestations, including vesicular/pompholyx/dyshidrotic, erosive/fissured, hyperkeratotic, or desquamative forms persisting for more than six months or recurring more than twice within a year. Subjective symptoms encompass sensations of pruritus, erythema, nociception, disruptions in sleep patterns, and alterations in mood [2].

For these reasons, it is a pathology often perceived as highly disabling, leading to significant stress and suffering and exerting a negative influence on work ability, career prospects, and social status.

Managing HE poses challenges due to the limited effectiveness and potential adverse effects associated with many currently available topical and systemic treatments. Dupilumab, a fully human monoclonal antibody, targets a shared receptor component involved in signaling interleukins IL-4 and IL-13. It has been approved by the FDA and EMA for treating conditions such as atopic dermatitis, asthma, chronic rhinosinusitis with nasal polyposis, eosinophilic esophagitis, and prurigo nodularis. This article explores the use of dupilumab in treating HE in adult patients. We present findings regarding twenty-one cases in which dupilumab was administered for the treatment of HE. All participants had previously experienced chronic and severe symptoms of HE and had tried multiple treatment approaches with limited success.

HE is a prevalent chronic condition that exerts a substantial and enduring adverse impact on quality of life (QoL) and imposes an economic burden on society. As already mentioned, the incidence of HE in the general population is 5–8%, with slightly higher incidence rates in women than in men (7.1 vs. 4.0) [2].

The pathogenesis of HE is multifactorial: on the one hand, there is a genetic component, while on the other, there is a role of the environment. Inherited factors inherent to an individual are likely associated with the functionality of the skin barrier. When the barrier is inherently weaker and more permeable than usual, it allows allergens and irritants to penetrate the skin, initiating localized immune responses. In fact, in atopic dermatitis (AD), there is a higher risk of developing HE since there is an underlying barrier defect primarily caused by mutations in some of its components, such as filaggrin and other proteins [3]. External factors contributing to HE encompass all influences that can degrade the skin barrier, including exposure to water or wet conditions, irritants, and mechanical friction on the skin. These factors can arise from occupational or domestic environments or a combination of both. Additionally, cold and dry weather, along with reduced indoor humidity, may also play a significant role.

HE is a condition involving inflammation of the skin, specifically on the hands, which can vary in cause, appearance, duration, and whether it is related to work or not. The classification of HE involves identifying specific subtypes based on these factors in order to better understand and treat individual cases. The classification of HE is divided into two categories based on the cause: exogenous, which includes irritant contact dermatitis (ICD), allergic contact dermatitis (ACD), and contact urticaria/protein contact dermatitis; and endogenous, which includes atopic HE, hyperkeratotic, and acute recurrent vesicular.

In clinical practice, categorizing HE is crucial for tailoring advice and treatment strategies for patients. Moreover, sub-diagnoses play a vital role in clinical trials by evaluating the efficacy of novel medications for HE.

The ESCD guidelines define the distinction between acute HE, which lasts for less than three months and occurs no more than once per year, and chronic HE, which lasts for more than three months or recurs two or more times per year [4]. In the acute stage of hand eczema, vesicles are commonly observed, while in the chronic stage, scaling and fissures are more predominant features.

It is important to acknowledge that sub-diagnoses of HE can evolve over time. If HE persists without resolution over several years, it warrants a re-evaluation. This involves repeating patch tests and investigating potential new causes. However, clinically, it is observed that chronic HE can reach a stage where it encompasses all subtypes, regardless of initial etiology or subtype. This end stage represents a state of self-sustaining chronic eczema.

**Figure 1:**
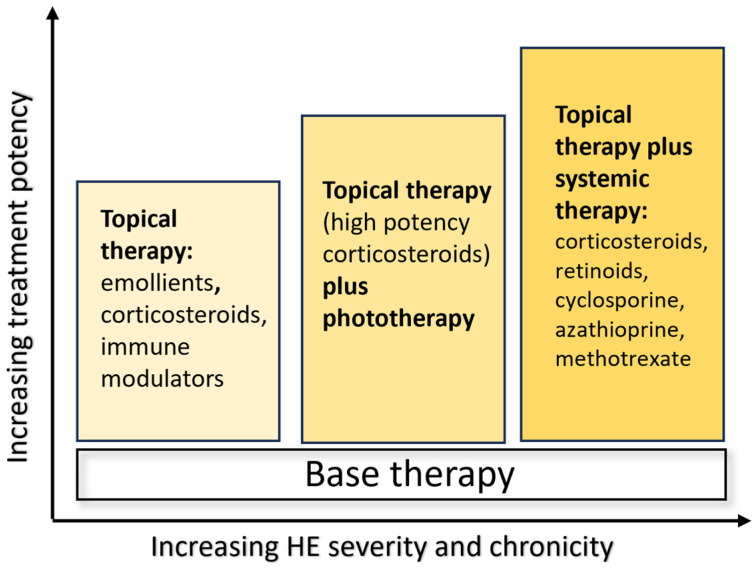
Treatment options for HE, depending on disease severity and chronicity [4].

Treatment options depend on the severity of the clinical manifestations, as reported in the ESCD guidelines, and include emollients, topical corticosteroids, calcipotriol and calcineurin inhibitors in mild–moderate disease, urea and ammonium lactate for hyperkeratotic variants, and systemic therapies with retinoids as alitretinoin and acitretin, and immunosuppressive drugs such as cyclosporine, azathioprine, methotrexate, and systemic corticosteroids in severe forms. Unfortunately, classical treatments are not always effective, and there are frequent recurrences, affecting many patients for years (Figure 1) [4].

## 2. Materials and Methods

### 2.1. Study Design

The primary objective of this investigation was to evaluate the effectiveness of dupilumab in managing individuals diagnosed with HE. The study encompassed adult patients aged 18 years and above undergoing monotherapy at the Dermatology Unit of the Polyclinic Tor Vergata Foundation in Rome, Italy. Data collection spanned from September 2019 to January 2024. Biologic-experienced patients within 12 weeks or patients treated with traditional therapies within four weeks were excluded. Patients who were using other topical treatments like corticosteroids were not included. Concerning topical treatment, only the use of topical emollients was permitted for patient enrollment in the study.

Patients were selected based on their medical records, which were reviewed by the doctors involved in the study. Dupilumab was administered to patients diagnosed with HE following the guidelines outlined in the Summary of Product Characteristics. This involved an initial induction phase with a subcutaneous injection of 600 mg at week 0, followed by a maintenance dose of 300 mg every two weeks thereafter. These guidelines were applied to patients who either did not respond to, or exhibited contraindications or adverse reactions to cyclosporine, as per Italian regulations. Diagnosis of HE relied on clinical manifestations. At enrollment, age, sex, body mass index (BMI), personal history of atopic dermatitis (AD) or other related conditions, age when HE started, duration of the disease, other health issues, and past and current treatments were recorded.

The disease severity was evaluated using various assessment tools: (a) the Hand Chronic Eczema Severity Index (HECSI), which has a range of 0 to 360; (b) the Itch Numeric Rating Scale (itch-NRS), scored from 0 to 10; (c) the Sleeplessness Numeric Rating Scale (sleep-NRS), also ranging from 0 to 10; and (d) the Dermatology Life Quality Index (DLQI), with scores ranging from 0 to 30. Before participating, all patients provided written consent, and the study followed ethical guidelines outlined in the 1975 Declaration of Helsinki. It is important to note that, according to Italian regulations, studies like this do not require formal approval from an ethics committee [5].

The effectiveness of dupilumab therapy was assessed by monitoring the HECSI, itch NRS, sleep NRS, and DLQI at baseline, week 6, and subsequently every 16 weeks thereafter. The endpoint considered was the reduction of HECSI by reaching HECSI 50, 75, 90, 100.

### 2.2. Study Population

The study comprised 21 adult participants, with a mean age of 43.5 years (SD 19.2); eleven were female. Among them, five individuals had a history of hypertension, with two also having diabetes. Additionally, two participants had a history of dyslipidemia, while one had Osler-Weber-Rendu Syndrome. Furthermore, one patient had a history of nasal polyposis, two had anxious-depressive syndrome, and another had autoimmune disease (Hashimoto thyroiditis). The average duration of the disease was 20 years (SD 16.5); seven patients had only HE, two had HE along with a previous history of AD, and twelve had HE along with active AD (two mild, five moderate, five severe). Regarding the type of HE, sixteen patients had a chronic fissured form, while five had a recurrent vesicular form. Among the participants, 11 individuals had a reported history of allergic rhinitis, five individuals had documented asthma, and an additional nine individuals had a history of allergic conjunctivitis as atopic comorbidities. Out of the cohort, eight patients yielded positive results in patch tests, with six showing positivity for nickel, two for cobalt, two for potassium bichromate, two for p-phenylenediamine, gum mix, methylisothiazolinone, and disperse blue, while one tested positive for balsam of Peru and one for fragrances. Seven patients tested positive for aeroallergens on skin prick tests.

Patients were previously treated with the following: 21 instances of topical corticosteroids, three applications of topical calcineurin inhibitors, four administrations of antihistamines, seven systemic corticosteroids, and eight instances of systemic drugs, which comprised either Cyclosporine (CyA) or Methotrexate (MTx) and acitretin (ACT). Additionally, one patient received phototherapy.

Baseline demographic and clinical information is presented in Table 1.

### 2.3. Outcome Measures

The efficacy of dupilumab for the treatment of HE was evaluated by measuring HECSI. HECSI scores were calculated at week 6, 16, 32, 52, 68, 84, 104, with a particular, focus on achieving HECSI 50 75, 90, and 100 (meaning a decrease in HECSI score of 50%, 75%, 90%, and 100%, respectively) during the observation period. HECSI is a score to assess the severity of HE by analyzing the intensity of six different clinical signs (erythema, induration/papulation, vesicles, fissuring, scaling, and edema) for five areas of the hand (fingertips, fingers (except the tips), palms, back of hands, and wrists). The score ranges from 0 to 360 for more severe forms [6].

Considering that patients in this study initiated treatment at different time points, the data presented should be regarded as a snapshot, offering a cross-sectional overview of our observations up to January 2024.

### 2.4. Statistical Analysis

Continuous variables are reported as mean  ±  standard deviation (SD), and categorical variables are reported as number and percentage. The values of EASI and HECSI are graphed, indicating at various time points the percentage of patients who reached HECSI 50, 75, 90, and 100. Missing values of intermediate visits were reported with the last observation carried forward (LOCF) method. *p* < 0.05 was considered for statistical significance. The analysis was conducted using SPSS (IBM SPSS Statistics for Windows, Version 27.0. IBM Corp, Armonk, NY, USA).

## 3. Results

A total of 21 patients diagnosed with HE were enrolled in this study. Data analysis was performed on the subset of these patients who received at least four doses of dupilumab over a treatment duration exceeding six weeks. Dupilumab demonstrated significant efficacy in substantially reducing multiple outcome measures, as evaluated by both medical practitioners and patients at each designated assessment timepoint (Week 6, Week 16, Week 32, Week 52, Week 68, Week 84, and Week 104), when compared to the baseline measurements.

The average HECSI score demonstrated a notable decrease from 161.2 (SD 56.3) at the beginning of the study to 14.0 (SD 22.8) by Week 16. This reduction was sustained, with a mean score of 0.0 (SD 0.0) maintained at Week 104. Similarly, the average itch-NRS score, reflecting one of the most distressing symptoms, decreased from 8.2 (SD 1.7) to 1 (SD 1.3) throughout the 104-week duration. This highlights a remarkable alleviation of itching symptoms, noticeable as early as Week 6, with an average score of 2.9 (SD 2.7).

The quality of sleep, evaluated using the mean sleep-NRS score, demonstrated improvement from an initial value of 5.1 (SD 3.2) to 0.0 (SD 0.0) by Week 104. Additionally, the mean DLQI score exhibited a significant decrease from 14.0 (SD 6.2) to 0.0 (SD 0.0) at Week 104, indicating a notable enhancement in dermatology-related quality of life. For the 12 patients with HE and active AD, dupilumab allowed disease clearance to be achieved, going from a mean EASI at a baseline of 20.8 (SD 9.2) to a mean EASI of 3.6 (SD 5.8) in just six weeks. This clearance was maintained at Week 104 with a mean score of 0.6 (SD 1.3).

With regard to the considered endpoint, it is evident that HECSI 50 was attained by 89.5% of patients at week 6 and by 100% at week 16, maintaining this achievement until week 104.

At week 6, HECSI 75 was achieved by 12 patients (57.9%). The proportion of patients meeting the HECSI-75 criteria steadily increased over the observation weeks, reaching 90% at week 16 and 100% at week 104.

**Figure 2:**
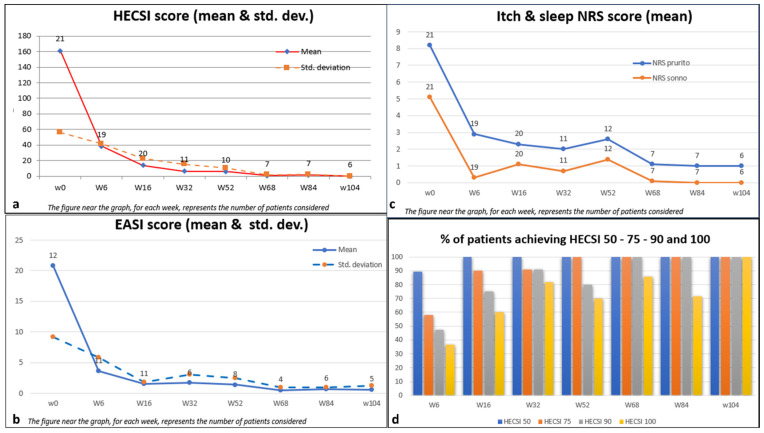
Effect of dupilumab on HECSI score, EASI score, and itch and sleep NRS over 104 weeks in patients with HE. Missing data for intermediate visits were filled in using the last observation carried forward (LOCF) technique. (**a**): mean HECSI scores in the overall population. (**b**): mean EASI scores in the overall population (**c**): mean itch and sleep NRS scores in the overall population (**c**): mean DLQI scores in the overall population (**d**): percentage of patients achieving HECSI 50, 75, 90, and 100 over 104 weeks. Statistical significance was observed in all time points considered (*p* < 0.05).

Furthermore, HECSI 90 and HECSI 100 were achieved by 75% and 60% of patients at week 16, and by 100% and 85% of patients at week 68, respectively. All patients who reached week 104 maintained complete disease remission according to HECSI 100. There were no dropouts; however, the number of patients decreased at each time point because some of them had not yet completed the full 104 weeks of evaluation. (Figure 2)

Despite the limitation imposed by the number of samples analyzed, no difference in response to treatment with dupilumab was observed among the various subtypes of HE and among patients with isolated HE compared to those with HE plus AD. Moreover, with regard to positive patch tests, we did not find any correlations with the response to treatment.

These findings underscore the efficacy of the intervention in ameliorating symptoms and promoting the overall well-being of the individuals involved in the study (Table 2).

Regarding the safety profile of dupilumab, the predominant adverse events reported in the literature consist of injection site reactions and cases of conjunctivitis. In our study, one patient encountered an episode of conjunctivitis, which was effectively treated solely with the application of eye drops.

## 4. Case Studies of Dupilumab Use in the Treatment of HE

We report three cases of patients affected by HE for years, resistant to conventional therapy, treated successfully with dupilumab.

### 4.1. Case 1

A 59-year-old woman with a history of hypertension, anxiety, hypercholesterolemia, and allergies to nickel, cobalt, and potassium bichromates, presents a palm–plantar condition marked by fissuring, erythema, and desquamation since 2007. No history of AD was reported by the patient. Prior therapeutic interventions included the administration of topical medications, oral corticosteroids, acitretin, methotrexate, IL-17 inhibitors, and IL-23 inhibitors, with the aim of addressing suspected pustulosis psoriasis, all of which proved unsuccessful.

In September 2019, a diagnosis of chronic hand and foot eczema was suspected, leading to the initiation of treatment with dupilumab. The HECSI was assessed at 270, while the itch NRS score, sleep NRS score, and DLQI were recorded as 10, 10, and 15, respectively.

**Figure 3:**
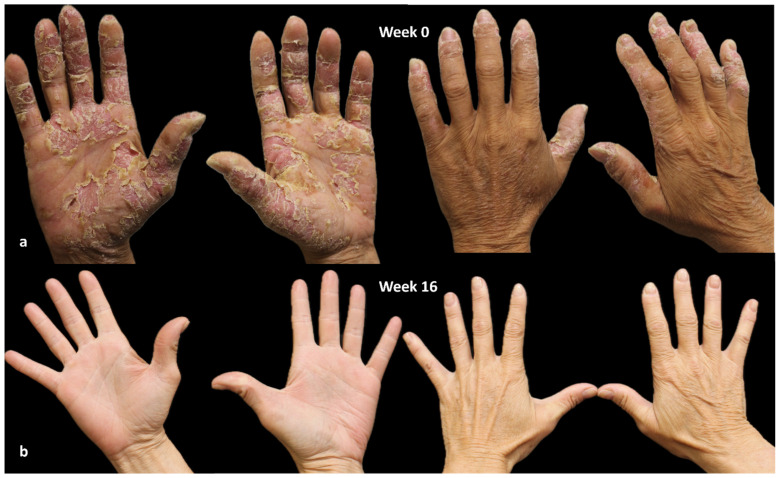
Chronic hand eczema treated with dupilumab. (**a**): Week 0 (HECSI 270); (**b**): week 16 (HECSI 0). Written consent for publication was obtained from the individual whose information is included in this study.

A notable response was achieved within the initial 16 weeks of treatment, marked by a notable reduction in disease indicators such as HECSI, itch and sleep NRS scores, and a substantial enhancement in quality of life, as indicated by a DLQI score of 0 (Figure 3).

The patient has been undergoing uninterrupted dupilumab treatment for a period of three years, and she remains in complete remission, with all indicators including HECSI, itch and sleep NRS, and DLQI scores equal to 0. She occasionally experiences episodes of conjunctivitis and periocular dermatitis. These episodes are treated with topical ointments and anti-inflammatory eye drops, showing a good response.

### 4.2. Case 2

A 60-year-old man with a history of IPB and respiratory allergies presented in January 2020 with severe palm–plantar eczema, previously treated with systemic corticosteroids and acitretin without improvement. Since 2016, he has been experiencing an intensely itchy palm–plantar condition, characterized by erythema, fissuring, desquamation, edema, and excoriation. The patient reported no manifestations of atopic dermatitis during his life.

The HECSI was assessed at 260, while the itch NRS score, sleep NRS score, and DLQI were recorded as 10, 7, and 17, respectively.

**Figure 4:**
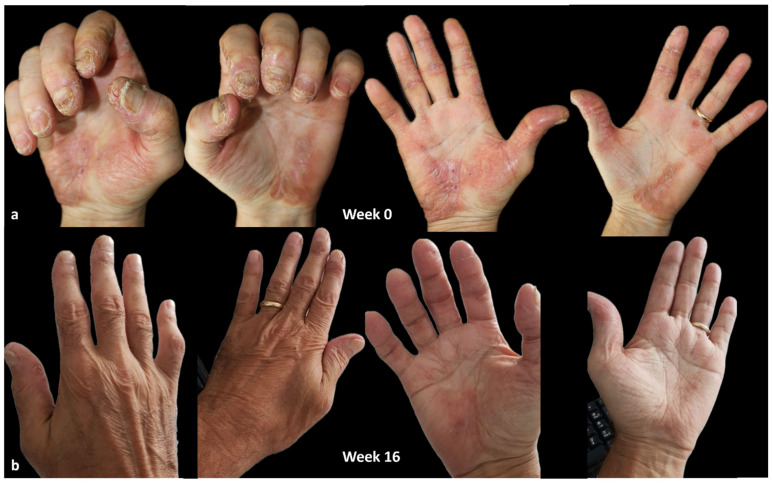
HE treated with dupilumab. (**a**): Week 0 (HECSI 260); (**b**): week 16 (HECSI 0). Written consent for publication was obtained from the individual whose information is included in this study.

In March 2020, treatment with dupilumab was initiated. A remarkable response was observed within the initial 16 weeks of treatment, characterized by the improvement in disease indicators such as HECSI, itch and sleep NRS scores, and a notable enhancement in quality of life (DLQI score of 0). The patient has been undergoing continuous treatment with dupilumab for a duration of three years, and he remains in complete remission, with all indicators including HECSI, itch and sleep NRS scores, and DLQI equal to 0 (Figure 4).

### 4.3. Case 3

A 22-year-old man, employed as a security guard, has been suffering from atopic dermatitis since the age of 20. He has a positive patch test history for various contact allergens. He presented in November 2022 with severe palm–plantar eczema, previously treated with topical and systemic corticosteroids with little benefit. Evaluation of HECSI was 230, and itch NRS score, sleep NRS score, and DLQI were 9, 8, and 25, respectively.

In December 2022, the administration of dupilumab commenced. A notable response was observed within the initial six weeks of treatment, marked by the improvement of disease indicators, including HECSI, itch and sleep NRS scores, and a significant enhancement in the quality of life (DLQI 0).

**Figure 5:**
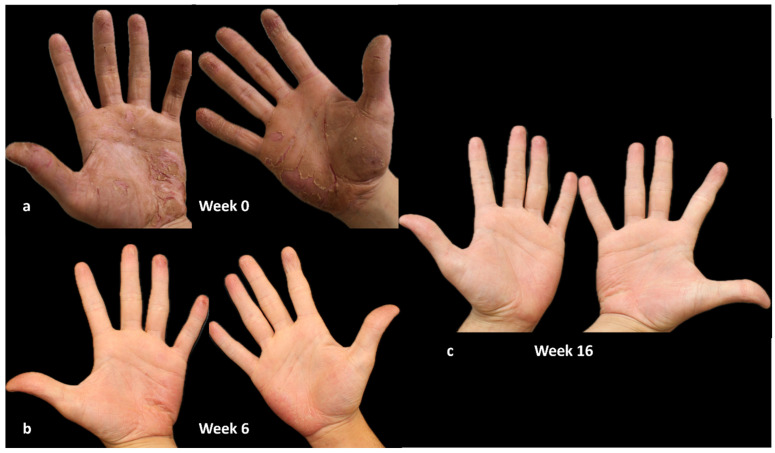
HE treated with dupilumab. (**a**): Week 0 (HECSI 230); (**b**): week 6 (HECSI 10); (**c**): week 16 (HECSI 0). Written consent for publication was obtained from the individual whose information is included in this study.

The patient is presently undergoing uninterrupted dupilumab treatment for one year, and he remains in complete remission, with all indicators including HECSI, itch and sleep NRS scores, and DLQI equal to 0 (Figure 5).

## 5. Discussion

HE presents significant challenges for both patients and physicians. Unlike many other medical conditions, there is a lack of large randomized controlled trials and officially approved treatment regimens specifically designed for HE.

Chronic eczema of the hands (CHE) is a condition that has a major impact on patients’ quality of life from both work and social perspectives. Patients are often forced to change jobs and habits because of the pain and burning associated with the lesions. To date, there are few medications available for the treatment of this condition. Among them is alitretinoin, which has a strong evidence base in the systemic treatment of HE and is approved for use in treating severe HE that does not respond or responds inadequately to topical corticosteroids.

Alitretinoin showed efficacy in a large observational open study as well as several RCTs at dosages of 10 or 30 mg/day. The use of alitretinoin may be limited by its high cost and side effect profile: myalgias, xerosis, cheilitis, alopecia, elevated serum lipids, headaches, and teratogenicity. Alitretinoin may not be a viable long-term treatment option for many patients [7,8].

Often, patients with severe chronic HE are difficult to treat and resistant to topical therapy alone, but they cannot undergo traditional systemic therapies continuously due to long-term side effects. For this reason, they require repeated cycles of therapy within the same year; for example, oral acitretin alternated with topical corticosteroids. Dupilumab, on the other hand, is a safe medication that can be used continuously without long-term adverse events. In fact, as in our experience, also in the study of J. Oosterhaven et al., in which 55 patients with AD and concomitant HE were treated with dupilumab, HECSI 75 was achieved by 28 (60%) at week 16. The mean HECSI score reduction was 49.2 points (range, 0–164; 95% within-subject confidence interval, 46.4–52.0), which was already significantly decreased after four weeks (*p* < 0.001). There was no difference in response between chronic fissured and recurrent vesicular clinical subtypes [9].

In their study, A.N. Voorberg et al. found that out of a total of 72 patients, HECSI 75 was achieved by 54 out of 62 patients (87.1%), and HECSI 90 was achieved by 39 out of 72 patients (62.9%) at 52 weeks. No disparity in response was observed among HE subtypes [10]. Even within our limited sample size, no variation in response to treatment with dupilumab was observed among the different subtypes of HE.

Conversely, in the case series by Olesen et al., dupilumab appears to be less effective in the group of patients with hyperkeratotic HE [11]. As already mentioned, in our experience, dupilumab yielded similar efficacy results across all subtypes of eczema analyzed.

This positive effect of dupilumab on HE has also been demonstrated in several case reports involving isolated, nonatopic HE, including vesicular HE, hyperkeratotic HE, ACD, and ICD [12,13]. Based on our study, it seems that there is no difference in the response to treatment with dupilumab between patients with isolated HE and those who have HE along with concurrent AD.

Supporting this finding is a study where the transcriptome of vesicular CHE was analyzed using RNA-sequencing, revealing a significant upregulation of the IL4R gene in lesional CHE skin compared to healthy control skin [14]. This indicates that the IL-4/IL-13 pathway may also contribute to isolated CHE.

In a recent multicenter phase 3 trial, 133 adults and adolescents (106/27) with moderate-to-severe hand and foot AD (43.6% of patients presented solely with hand AD, while 3.0% had foot AD exclusively) were evaluated after a 16-week treatment with dupilumab. Results showed that a significantly higher proportion of patients receiving dupilumab achieved the primary endpoint of reaching an IGA score of 0 or 1 for hand and foot lesions (HFIGA 0/1) by week 16 compared to those on placebo (40.3% vs. 16.7%; *p* = 0.003). The distinction between the two groups was noticeable from as early as week 4 and persisted until the end of the 16-week period. Moreover, a greater number of dupilumab-treated patients achieved a key secondary endpoint of experiencing a reduction of at least four points in the Hand and Foot Peak Pruritus NRS (HF-Peak Pruritus NRS) from baseline at week 16 compared to those on placebo (52.2% vs. 13.6%; *p* < 0.0001). This disparity in outcomes between the dupilumab and placebo groups was evident as early as week 1 and continued through week 16. Furthermore, HECSI 75 was reached by 46.9% vs. 21.5% (*p* < 0.01) of patients at week 16 [15]. This recent experience provides further confirmation of our findings regarding the effectiveness of dupilumab in treating HE, albeit constrained by a very brief observational period. In our case, the benefits observed in controlling HE extend up to two years of observation.

Additional treatments being evaluated for HE include Janus kinase inhibitors (JAKis). For example, Delgocitinib is a topical small molecule pan-JAK inhibitor with activity against JAK1, JAK2, JAK3, and TYK2. Topical delgocitinib 0.5% ointment is approved in Japan for the treatment of AD in adults, and the FDA has granted Fast Track Designation for the treatment of adult moderate-to-severe CHE [16]. In terms of novel systemic treatments, a recent phase 2b clinical trial assessed the efficacy of gusacitinib, an oral drug that functions as a small molecule inhibitor targeting the pathways of spleen tyrosine kinase (SYK) and Janus kinases (JAK 1, 2, 3, and TYK2). This mechanism effectively suppresses the signaling of Th17, Th1, Th2, and Th22 cells [17].

JAKis such as upadacitinib and abrocitinib were evaluated in post hoc analyses of phase 3 trials involving generalized AD with concurrent HE. However, there is currently no efficacy data available for HE treated separately. In the Measure Up 1 and 2 trials, upadacitinib monotherapy demonstrated clinically significant enhancements in HECSI scores at week 16 among adults with mild-to-severe HE [18]. Baricitinib has also been used successfully in the treatment of severe hand eczema, as reported by some case reports [19]. In contrast to dupilumab, which functions as a targeted immunomodulator, JAKis are regarded as broad immunosuppressants. Their administration is associated with a heightened susceptibility to infections and necessitates routine laboratory monitoring for conditions such as hyperlipidemia and cytopenias. In summary, treatment with dupilumab alone led to prompt, clinically significant, and statistically notable improvements of HE.

## 6. Conclusions

HE heavily impacts individuals’ QoF. The associated treatment expenses, along with indirect societal costs such as reduced work capacity, sick leave, and job displacement, are substantial. Given the significant negative consequences for QoF and the direct and indirect costs to society, there is a need for efficient and safe treatment. In this panorama, dupilumab, a biological drug with an excellent safety profile, appears to be the best choice.

As in some experiences already mentioned in the scientific literature, in our study as well, dupilumab was shown to be an effective drug in achieving disease clearance, as indicated by all the parameters considered (HECSI, itch NRS, sleep NRS, DLQI) at each evaluation point (Week 6, Week 16, Week 32, Week 52, Week 68, Week 84, and Week 104), in comparison to the initial baseline.

## Figures and Tables

**Table 1:** Demographic and clinical characteristics.

Demographic	
Total population	21
Age (mean ± SD)	43.5 ± 19.2
M/F	10/11
BMI (mean ± SD)	24.1 ± 4
Comorbidities	
Hypertension	5
Diabetes	2
Dyslipidemia	2
Osler-Weber-Rendu Syndrome	1
Nasal polyposis	1
Autoimmune disorder (Hashimoto thyroiditis)	1
Anxious-depressive syndrome	2
Course of disease	
Duration of disease (mean ± SD)	20 ± 16.5
HE only	7
Elevated IgE	4
HE and previous AD	2
Elevated IgE	2
HE and active AD	12
Elevated IgE	8
Severity of AD	
- Mild - Moderate - Severe	2 5 5
Type of HE	
- Chronic fissured Elevated IgE - Recurrent vesicular Elevated IgE	16 11 5 3
Allergic comorbidities	
Allergic rhinitis	11
Asthma	5
Allergic conjunctivitis	9
Prick test positive, N (%)	7 aereoallergens (33.3%)
Patch test positive, N (%)	8 (38.1%)
- Nichel - Cobalt - Potassium bichromate - P-phenylenediamine, gum mix, methylisothiazolinone, disperse blue - Balsam of Perù - Fragrances	6 2 2 2 1 1
Previous treatments	
Topical Corticosteroids	21
Topical Calcineurin Inhibitors	3
Antihistamines	4
Systemic Corticosteroids	7
Systemic Drugs (CyA o MTx o ACT)	8
Phototherapy	1

**Table 2:** Results.

	Overall Population							
	Baseline	Week 6	Week 16	Week 32	Week 52	Week 68	Week 84	Week 104
HECSI score (mean ± SD)	161.2 ± 56.3	38.7 ± 41.6	14.0 ± 22.8	6.4 ± 15.7	6.0 ± 10.7	0.7 ± 1.9	1.4 ± 2.4	0.0 ± 0.0
EASI score (mean ± SD)	20.8 ± 9.2	3.6 ± 5.8	1.5 ± 1.8	1.7 ± 3.1	1.4 ± 2.5	0.5 ± 1	0.7 ± 1.0	0.6 ± 1.3
itch-NRS score (mean ± SD)	8.2 ± 1.7	2.9 ± 2.7	2.3 ± 2.7	2 ± 2.6	2.6 ± 3.3	1.1 ± 1.3	1.0 ± 1.3	1.0 ± 1.3
sleep-NRS (mean ± SD)	5.1 ± 3.2	0.3 ± 0.7	1.1 ± 2.1	0.7 ± 2.4	1.4 ± 3.3	0.1 ± 0.4	0.0 ± 0.0	0.0 ± 0.0
DLQI score (mean ± SD)	14 ± 6.2	2.2 ± 2.7	1.5 ± 2.5	1.4 ± 2.0	1.2 ± 2.6	0.3 ± 0.5	0.6 ± 1.5	0.0 ± 0.0
N. (%) patients reaching HECSI 50		89.5%	100%	100%	100%	100%	100%	100%
N. (%) patients reaching HECSI 75		57.9%	90%	90.9%	100%	100%	100%	100%
N. (%) patients reaching HECSI 90		47.4%	75%	90.9%	80%	100%	100%	100%
N. (%) patients reaching HECSI 100		36.8%	60%	81.8%	70%	85.7%	71.4%	100%

## Data Availability

The original contributions presented in the study are included in the article; further inquiries can be directed to the corresponding author.
